# Representational recoding and capacity limits: a conceptual reinterpretation of Sidney Smith’s experiment

**DOI:** 10.3389/fncom.2026.1878543

**Published:** 2026-08-11

**Authors:** Mikhail Inyushin

**Affiliations:** Universidad Central del Caribe School of Medicine, Bayamón, PR, United States

**Keywords:** chunking, neuromorphic computing, representation learning, Sidney Smith, vector representations, working memory

## Abstract

George A. Miller’s classic discussion of memory capacity and Sidney Smith’s recoding experiments demonstrated that cognitive limits depend more strongly on the number of active representational units (“chunks”) than on the total amount of raw information being processed. Here, we reinterpret Smith’s experiments from the perspective of modern computational neuroscience and representation learning. We argue that Smith’s recoding procedure illustrates a general principle of representational recoding, whereby learning increases the amount of information associated with each active representational unit without increasing the number of units available to the system. This principle provides conceptual links among classical theories of chunking, modern representation learning, vector symbolic architectures, and biological memory systems. The framework may offer useful guidance for future neuromorphic systems operating under strict energetic and structural constraints.

## Introduction

The classic demonstration experiment reported by Sidney Smith before the Eastern Psychological Association in 1954, preserved primarily through George A. Miller’s influential review The Magical Number Seven, Plus or Minus Two, provides an early and remarkably prescient insight into the nature of cognitive capacity limits and their circumvention through representational change (Miller, 1956). Although originally framed in the language of immediate memory and information theory, Smith’s experiment can also be viewed from the perspective of modern representation learning as an early illustration of how learning changes representational format to increase effective capacity under fixed resource constraints.

Smith began with a well-established empirical observation: human immediate memory can retain approximately seven to nine discrete items, whether those items are digits, letters, or other simple symbols (Miller, 1956). Subsequent work has suggested that effective working-memory capacity is often closer to four chunks under controlled experimental conditions (Cowan, 2001). Regardless of the precise number, both perspectives emphasize a common principle: cognitive performance depends strongly on the number of simultaneously active representational units.

Smith hypothesized that if memory is constrained primarily by the number of active representational units rather than by the total number of bits being represented, then effective capacity should be increased by recoding low-information symbols into higher-information units.

To test this idea, Smith taught subjects explicit recoding schemes that grouped binary digits into larger symbolic units. One of the simplest examples involved recoding pairs of binary digits into base-4 symbols (Table 1).

**TABLE 1:** Example of binary-to-base-4 recoding.

Binary pair	Recoded symbol
00	0
01	1
10	2
11	3

Under this transformation, a sequence of binary digits can be represented using fewer symbolic units while preserving all original information. For example (Table 2).

**TABLE 2:** Example of representational recoding.

Binary sequence	Recoded representation
00011011	0123

In this example, eight binary digits are represented by four base-4 symbols. No information is lost, but the number of active representational units is reduced by half. Smith also employed larger groupings using octal and decimal recoding schemes, allowing progressively more information to be associated with each remembered unit. Following training, subjects recalled binary sequences while silently applying these learned transformations. Even with relatively little practice, memory span increased substantially. With extensive training, Smith’s own performance approached theoretical limits, allowing recall of approximately forty binary digits without error (Miller, 1956).

The key result of Smith’s experiment is not merely that mnemonic strategies can extend memory span. Rather, it suggests that cognitive capacity depends strongly on the number of active representational units and that learning can increase effective capacity by altering how information is represented within those units. In modern terms, memory performance may depend not only on the amount of information available to a system but also on the representational efficiency with which that information is organized.

From a computational perspective, Smith’s recoding procedure can be understood as a form of representational recoding. Raw binary inputs are transformed into fewer, higher-information symbolic units via learned encoding rules. Importantly, the information itself is not compressed in the strict information-theoretic sense because it remains recoverable. Instead, the amount of information associated with each active representational unit increases.

This interpretation aligns naturally with modern representation learning. Representation learning refers to methods that automatically discover useful internal representations of data rather than relying exclusively on manually constructed features (Bengio et al., 2013). Although Smith’s procedure differs substantially from contemporary machine-learning algorithms, both approaches exploit a common principle: performance can often be improved by changing representational format rather than by increasing available storage resources.

Contemporary neural networks routinely transform discrete symbols into distributed internal representations that capture semantic, structural, and statistical regularities relevant to the task being learned. A word embedding, for example, is a learned vector representation in which similar words acquire similar numerical representations because they are used in similar contexts. While Smith’s recoded symbols are not vector embeddings in the modern machine-learning sense, both approaches illustrate how learning can increase the amount of task-relevant structure associated with each active representation, thereby improving the effective use of limited computational resources.

Furthermore, Smith’s observation that recoding must become automatic anticipates an important aspect of many learning systems. Initially, recoding requires conscious effort and explicit application of learned rules. With practice, these transformations become increasingly automatic. This distinction suggests a potentially important difference between Smith’s paradigm and many modern representation-learning approaches. Whereas contemporary machine-learning systems often learn internal representations automatically through optimization, Smith’s subjects first learned explicit symbolic transformations that were subsequently automatized through practice.

Viewed through this lens, Smith’s experiment can be interpreted as an early conceptual demonstration of representational recoding under fixed-capacity constraints. Memory span corresponds approximately to a limited number of active representational units, while recoding increases the task-relevant information associated with each unit without increasing their number. This perspective also helps explain why expertise, language, and abstraction can dramatically expand apparent cognitive capacity: they enable richer and more efficient representations to occupy the same limited set of active memory slots (Chase and Simon, 1973; Gobet et al., 2001; Lupyan and Bergen, 2016).

## Formalization as a principle of representational recoding

Let K denote the number of simultaneously active representational units available to a cognitive or computational system. In Smith’s original experiment, a representational unit corresponds to a consciously maintained symbolic chunk, whereas in modern computational systems it may correspond to an embedding, a latent feature, a symbolic structure, or another task-relevant representation. Let I denote the average amount of information per active unit. Effective memory capacity depends jointly on these two factors:


C∝K⁢I

where C represents effective capacity. In Smith’s original experiment, I corresponds approximately to the amount of symbolic information represented by each consciously maintained chunk and may be expressed in bits per chunk. In modern distributed representations, however, I should be interpreted more generally as representational efficiency, reflecting the amount of task-relevant information encoded by each active representation. Depending on the computational framework, this may arise from embedding structure, distributed feature encoding, or high-dimensional vector representations.

Smith’s experiment demonstrates that learning can increase effective capacity by increasing I without increasing K. Recoding, therefore, does not increase the number of active representational units. Instead, it increases the amount of information associated with each active unit by transforming information into a more efficient representational format.

This formulation suggests that apparent increases in memory capacity may often reflect changes in representation rather than changes in the underlying storage resources available to the system.

Working-memory limitations have also been reported in great apes, although the estimated capacity is substantially smaller than that typically observed in humans (Read et al., 2022). Nevertheless, these findings suggest that limitations on the number of simultaneously active representations may reflect a broadly conserved property of primate cognition while allowing important quantitative differences between species.

## Implications for neural network training

Modern neural networks already exploit this principle extensively. Embedding layers, latent spaces, bottleneck architectures, and attention mechanisms all modify the representational format to improve performance under resource constraints. Transformers, for example, operate with finite context windows yet achieve remarkable expressive power through learned internal representations and hierarchical abstraction (Vaswani et al., 2017).

The concept of representational recoding bears strong similarities to representation learning. Deep neural networks progressively transform raw sensory inputs into internal representations that capture statistical regularities relevant to the learning objective or downstream task (Bengio et al., 2013). Depending on the architecture and training objective, these representations may become more compact, more structured, or more useful for subsequent computation, while the degree of redundancy retained varies across learning methods. In this respect, Smith’s experiment may be viewed as an early demonstration that effective information capacity can be increased through changes in representational format rather than through expansion of storage resources.

A particularly interesting parallel exists with autoencoders and bottleneck architectures. These systems learn encoder-decoder mappings that transform inputs into lower-dimensional latent representations while preserving essential information required for reconstruction (Hinton and Salakhutdinov, 2006). Smith’s behavioral recoding procedure is not identical to such approaches. Nevertheless, both illustrate a common computational principle: effective processing can often be improved by learning more efficient representations rather than by increasing available resources.

Importantly, Smith’s procedure differs from most contemporary representation-learning approaches in one significant respect. Modern neural networks typically learn internal representations automatically through optimization of task-specific objectives, whereas Smith’s subjects consciously acquired explicit recoding rules that gradually became automatic through practice. This distinction suggests the existence of an intermediate class of learning systems that combine deliberate symbolic restructuring with subsequent automatization. Such mechanisms remain relatively underexplored in both artificial intelligence and computational neuroscience.

## Vector Symbolic Architectures and distributed representations

A related connection exists with Vector Symbolic Architectures (VSAs), a family of computational frameworks that represent information using high-dimensional distributed vectors (Kanerva, 2009; Plate, 1995; Eliasmith, 2013; Kleyko et al., 2023). Although individual VSA approaches differ substantially in their mathematical implementation, many employ operations such as superposition and binding to represent multiple items within fixed-width vectors while preserving the ability to recover relevant information.

The primary appeal of VSAs is that they provide a framework for representing structured information in a distributed manner while retaining many properties of symbolic reasoning. VSA models have been applied to memory, analogy, reasoning, sequence processing, and cognitive architectures. Rather than relying on increased storage resources, many VSA approaches aim to improve computational efficiency by transforming the representation format.

Although Smith’s behavioral recoding procedure and modern Vector Symbolic Architectures differ substantially in their implementation, both embody representational principles illustrating how reorganizing information can improve the use of limited computational resources. In each case, information is reorganized into representations that permit more effective use of limited computational resources. Smith’s experiment can therefore be viewed as an early illustration of a broader principle that later emerged in several computational approaches to cognition: changes in representational format may increase effective capacity without requiring proportional increases in underlying resources.

## Biological constraints and future directions

The principles revealed by Smith’s experiment may also provide a useful conceptual framework for understanding hippocampal mnemonic functions. Smith demonstrated that memory performance can be increased not by expanding the number of active memory units, but by transforming information into more efficient representational formats through learned recoding. A conceptually similar principle may operate in the hippocampus. Computational and experimental studies suggest that the dentate gyrus primarily supports pattern separation, whereas recurrent circuitry in CA3 plays an important role in associative recall and pattern completion. However, these functions are not completely segregated, and evidence indicates substantial functional overlap among the dentate gyrus, CA3, and CA1 during memory encoding and retrieval (Marr, 1971; McClelland et al., 1995; Yassa and Stark, 2011). From the perspective proposed here, these processes can be viewed as biologically plausible forms of representational recoding, in which neural activity is reorganized into sparse, distinctive patterns that improve retrieval efficiency while minimizing metabolic cost. A plausible biological mechanism underlying such representational transformations is the sparse coding strategy of the dentate gyrus. Only a small fraction of dentate granule cells is active during the encoding of a given experience, producing highly selective neural representations that reduce interference between similar memories while minimizing metabolic expenditure (Leutgeb et al., 2007; Yassa and Stark, 2011). These sparse activity patterns are subsequently associated through the recurrent collateral network of CA3, allowing partial cues to reconstruct complete memory representations during retrieval (Marr, 1971; McClelland et al., 1995). Rather than increasing the number of simultaneously active representations, this organization increases the distinctiveness and informational content of each representation, thereby improving effective representational capacity. In this respect, hippocampal circuitry illustrates how biological neural systems may enhance effective representational capacity while operating under strict energetic and structural constraints. Smith’s experiment, therefore, suggests that an important function of memory systems may not simply be storage itself, but the transformation of information into representations that maximize effective capacity while preserving recoverability.

The analogy between chunking and representation learning also raises important questions concerning biological learning. Unlike many artificial systems that can rely on extensive optimization before deployment, biological organisms must learn while continuously interacting with their environment. Although computational cost depends on many factors, including architecture, parameter count, and implementation, biological systems face the additional constraint that learning and inference must occur during ongoing behavioral interaction with the environment. Consequently, representational transformations in biological systems must emerge under constraints of limited energy, finite time, and ongoing behavioral demands. Local learning mechanisms, including Hebbian learning (Hebb, 1949) and spike-timing-dependent plasticity (Bi and Poo, 1998; Caporale and Dan, 2008), may contribute to such transformations, although the present argument does not depend on any specific biological learning rule.

An alternative interpretation of working-memory limitations is that they represent an adaptive compromise between representational efficiency and processing speed. Smith’s experiments demonstrated that effective memory capacity could be increased through recoding, but only at the cost of additional processing that initially required conscious effort and practice. Increasing the complexity of recoding can expand effective capacity, yet it also introduces computational demands that may delay behavioral responses. Because biological organisms must operate continuously in real time, natural selection may favor representational strategies that sacrifice some storage efficiency in exchange for rapid processing. From this perspective, limitations on the number of active representations may not simply reflect resource constraints, but may instead constitute an optimization balancing memory capacity, computational cost, and reaction speed.

The distinction between explicit and automatic recoding suggests several promising directions for future research. Contemporary representation-learning systems typically focus on automatically acquiring internal representations through optimization. Smith’s experiments, however, demonstrate a different sequence: explicit symbolic transformations are first learned and subsequently become automatic through practice. Future intelligent systems may benefit from learning not only representations themselves, but also explicit recoding strategies that can later be transformed into efficient automatic processing routines. This possibility suggests a developmental sequence in which intelligent systems first acquire explicit symbolic transformations and subsequently compile them into automatic representational operations, potentially providing a bridge between classical cognitive theories of chunking and modern representation-learning frameworks.

Additional opportunities arise in neuromorphic engineering. If effective capacity depends jointly on the number of active representations and the information associated with each representation, then future architectures may benefit from optimizing representational density rather than simply increasing parameter count. Such systems may be particularly valuable in environments where computational resources, energy consumption, or communication bandwidth are constrained.

## Conclusion

Sidney Smith’s (1954) demonstration is not merely a historical curiosity. It illustrates a general principle of intelligent systems: effective capacity depends not only on the number of representational units available to a system, but also on the efficiency with which information is represented within those units. By learning new representational formats, cognitive systems can increase effective capacity without increasing underlying storage resources.

Viewed from this perspective, Smith’s experiments provide an early conceptual demonstration of representational recoding under fixed-capacity constraints. Although the specific mechanisms differ substantially across the systems discussed here, a common theme is that performance depends not only on the amount of information available but also on how that information is represented. This principle may help unify classical observations from cognitive psychology with contemporary developments in artificial intelligence, computational neuroscience, and neuromorphic engineering.
